# Runs of homozygosity and inbreeding in thyroid cancer

**DOI:** 10.1186/s12885-016-2264-7

**Published:** 2016-03-16

**Authors:** Hauke Thomsen, Bowang Chen, Gisella Figlioli, Rossella Elisei, Cristina Romei, Monica Cipollini, Alfonso Cristaudo, Franco Bambi, Per Hoffmann, Stefan Herms, Stefano Landi, Kari Hemminki, Federica Gemignani, Asta Försti

**Affiliations:** Molecular Genetic Epidemiology, C050, German Cancer Research Center (DKFZ), Im Neuenheimer Feld 580, 69120 Heidelberg, Germany; Department of Biology, University of Pisa, Pisa, Italy; Department of Endocrinology and Metabolism, University of Pisa, Pisa, Italy; Blood Centre, Azienda Ospedaliero Universitaria A. Meyer, Firenze, Italy; Department of Genomics, Life and Brain Center, University of Bonn, Bonn, Germany; Division of Medical Genetics, University Hospital Basel, Basel, Switzerland; Department of Biomedicine, University Hospital Basel, Basel, Switzerland; Center for Primary Health Care Research, Clinical Research Center, Lund University, Malmö, Sweden

**Keywords:** Thyroid cancer, Runs of homozygosity, Inbreeding, GWAS

## Abstract

**Background:**

Genome-wide association studies (GWASs) have identified several single-nucleotide polymorphisms (SNPs) influencing the risk of thyroid cancer (TC). Most cancer predisposition genes identified through GWASs function in a co-dominant manner, and studies have not found evidence for recessively functioning disease loci in TC. Our study examines whether homozygosity is associated with an increased risk of TC and searches for novel recessively acting disease loci.

**Methods:**

Data from a previously conducted GWAS were used for the estimation of the proportion of phenotypic variance explained by all common SNPs, the detection of runs of homozygosity (ROH) and the determination of inbreeding to unravel their influence on TC.

**Results:**

Inbreeding coefficients were significantly higher among cases than controls. Association on a SNP-by-SNP basis was controlled by using the false discovery rate at a level of q* < 0.05, with 34 SNPs representing true differences in homozygosity between cases and controls. The average size, the number and total length of ROHs per person were significantly higher in cases than in controls. A total of 16 recurrent ROHs of rather short length were identified although their association with TC risk was not significant at a genome-wide level. Several recurrent ROHs harbor genes associated with risk of TC. All of the ROHs showed significant evidence for natural selection (iHS, F_st_, Fay and Wu’s H).

**Conclusions:**

Our results support the existence of recessive alleles in TC susceptibility. Although regions of homozygosity were rather small, it might be possible that variants within these ROHs affect TC risk and may function in a recessive manner.

**Electronic supplementary material:**

The online version of this article (doi:10.1186/s12885-016-2264-7) contains supplementary material, which is available to authorized users.

## Background

Thyroid cancer (TC) is the most common malignancy of the endocrine system with incidence rates being 2 to 3 times higher in women compared with men [1, 2]. In economically developed countries, 0.5 to 10 TC cases are diagnosed per 100 000 individuals each year [1]. Significant regional differences are seen in Europe with Italy being among the countries with the highest incidence rates in the world (Cancer Incidence in Five Continents, IX, 2000, http://www.iarc.fr/en/publications/pdfs-online/epi/sp160/). While exposure to ionizing radiation or insufficient iodine intake is an established risk factor, anthropometric risk factors such as high body surface area, great height, or excess weight have been associated with increased TC risk [3]. However, TC is also characterized by having one of the highest familial risks of any cancer supporting heritable predisposition [4–6]. A high risk of TC is associated with some genetic disorders, but most of the familial risk of TC remains unexplained [7]. During the last years genome-wide association studies (GWASs) have provided robust evidence for common susceptibility to TC. At least four GWASs have identified a set of genes with susceptibility for TC [8–11]. These studies suggest that much of the familial risk of TC may be due to the coinheritance of multiple low/moderate-penetrant alleles, some of which may be common. The majority of cancer predisposition genes identified through the GWASs function in a co-dominant manner, and no evidence has been found for recessively functioning disease loci in TC, although the risk for TC among siblings is much higher than the parent-offspring risk, suggesting recessive inheritance [6]. Recessive inheritance has been associated with consanguinity or an increased risk in populations characterized by a higher degree of inbreeding and corresponding homozygosity [12]. A consecutive pattern, called runs of homozygosity (ROH), appears mainly in an increased frequency due to a high level of relatedness between individuals within a population or due to selection [13]. These ROHs are shown to predispose to many genetic diseases including cancers [14–16]. The siblings-risk and the fact and that TC is part of recessively inherited syndromes such as the Werner syndrome make TC an ideal target to search for recessively acting disease loci [6, 7].

In a first step we estimated the proportion of the total phenotypic variance explained by all common SNPs for TC risk. This was followed by a whole-genome homozygosity analysis based on our previous GWAS in the high-incidence Italian population. The aim of our study was to examine whether inbreeding or homozygosity is associated with an increased risk of TC and to search for novel recessively acting disease loci.

## Methods

### Ethics statement

Study participants were recruited according to the protocols approved by the institutional review boards in accordance with the Declaration of Helsinki. All subjects provided written informed consent. This study was approved by the ethics committees of the University Hospitals of Cisanello and Santa Chiara in Pisa, Italy and of the Meyer Hospital in Florence, Italy.

### Genomic data - quality control of SNP genotyping

The study is based on the genotyping data of our previously performed GWAS on the Italian cases and controls, and did not include any new participants [11, 17]. All patients were ascertained with papillary thyroid cancer (PTC) through the University Hospital Cisanello in Pisa. After a stringent quality control procedure the final set consisted of 649 cases and 431 controls with genotype data on 536 270 SNPs [18, 19]. Data have been submitted to a central database: www.gwascentral.org.

### Proportion of the total phenotypic variance explained by all common SNPs

The approach of Yang et al. was used to estimate the proportion of the total phenotypic variance explained by all common SNPs [20]. First, we estimated the genetic relationship matrix (GRM) for each individual autosome of all the individuals and fitted the GRMs in a mixed linear model (MLM) to estimate the proportion of the phenotypic variance explained by all common SNPs. We repeated this scenario after excluding 15 identified GWAS regions for TC including the genomic region 500 kb upstream and downstream [11, 17]. This left us with a total of 520 137 autosomal SNPs.

For both scenarios sex and eigenvectors from 10 principal components of the population structure were used as covariates. Consecutive estimates on the observed 0–1 scale are linearly transformed to that on the unobserved continuous liability scale such that h_l_^2^ = h_0_^2^K(1 − K)/z^2^ [21], where K is the prevalence of the disease and z is the value of the standard normal probability density function at the threshold t. Given an incidence of 8 – 9/100 000/year will result in a cumulative risk of ~ 6 in 1000 as an estimate of the prevalence. Estimation was performed using restricted maximum likelihood (REML) via the genome-wide complex trait analysis (GCTA) software [22].

### Genome-wide assessment of associations between homozygosity at individual SNPs and TC

A chi^2^-test was performed to test for any association between homozygosity and susceptibility of TC on a SNP-by-SNP basis in our entire sample series [14]. To control the problem of multiple testing the false discovery rate (FDR) was calculated and controlled at an arbitrary level q* < 0.05 [23].

### Statistical and bioinformatics analysis

We defined ROHs following recommendations in Howrigan et al. [24] ROHs were detected using PLINK (v1.07) software. To prevent overestimating the number and size of ROHs no heterozygous SNPs were permitted in any window. We kept the remaining options to default values. The parameter for the “homozyg-kb” option was also kept at the default value of 1000 kb to select individual segments of minimal length. We only varied the parameter “homozyg-snp” option according to the definition of ROHs as below. Subsequent statistical analyses were performed using packages available in the R statistics package [25]. Comparison of the distribution of categorical variables was performed using the chi^2^-test. To compare the difference in the average number of ROHs between cases and controls, we used the Student’s *t*-test. Naive adjustment for multiple testing was based on the Bonferroni correction.

### Identification of homozygosity

We used the method of Lencz et al. to estimate the minimum number of consecutive homozygous SNPs required to form a ROH that was more than an order of magnitude larger than the mean haploblock size in the human genome without being too large to be very rare [26]. In our TC data, with 1080 individuals and 536 270 SNPs, the mean heterozygosity in controls was calculated to be 35 %. Thus, a minimum length of 53 would be required to produce <5 % randomly generated ROHs across all subjects: ((1–0.35)^53^ × 536 270 × 1080 ≤ 0.05). Due to linkage disequilibrium (LD) between the SNPs, the SNP genotypes are not always independent. Pairwise LD was estimated using the SNP pruning function of PLINK, with a default value of *r*^*2*^ > 0.8 and restricting the search of tagging SNPs within each 250 kb window. Approximately 377 000 separable tag groups were discovered, representing an >25 % reduction of information compared with the original number of SNPs. Thus, ROH length of 75 was used to approximate the degrees of freedom of 53 independent SNP calls.

The R statistics package was used to identify a list of ‘common’ ROHs with 75 consecutive homozygous SNP calls across a certain number of samples and with each ROH having identical start and end locations across the individuals. The “homozyg-group” option of the PLINK package was used to produce a file of the overlapping ROHs separated into pools containing the number of cases and controls carrying the ROH. We considered pools with more than five samples and at least 500 kb of length as recurrent ROHs. A consensus SNP set representing the minimal overlapping region across all samples in the pool was used to define the recurrent ROHs. The association of the recurrent ROHs was then tested for differences of the average proportion of ROHs among cases and controls. Within each overlapping ROH the proportion of homozygous genotypes at each SNP was calculated for cases and controls separately, and the significance of the difference was tested by a one-tailed *t*-test.

### Testing the effects of natural selection

We used three metrics, the integrated haplotype score (iHS), the fixation index (F_st_) and Fay and Wu’s H to investigate the selective pressure due to demographic events (e.g. bottleneck events, founder effects or population isolation) on each recurrent ROH [27, 28]. All metrics were obtained from Haplotter Software (University of Chicago, Chicago, IL, USA; http://haplotter.uchicago.edu/) [28, 29].

### Testing the effects of inbreeding

To test whether inbreeding influenced the susceptibility to TC, three different inbreeding coefficients (F I, F II and F III) were derived for each individual based on their SNP data using GCTA [22]. The coefficients were tested for differences between cases and controls using a Student’s *t*-test. We also used a generalized linear regression model (GLM) and regressed F I, F II or F III as explanatory variables on the disease status of the TC patient as the binary response (0/1). We included several covariates in the model: the sex of the individuals, the first 10 ancestry-informative principal components and the percentage of SNPs missing for an individual.

A genomic measure of individual homozygosity (F_ROH_) was calculated by a method proposed by McQuillan et al. [30] in which L_ROH_ is the sum of ROHs per individual above a certain criterion length (i.e. 1000 kb as defined beforehand) and L_AUTO_ is the total SNP-mappable autosomal genome length, excluding the centromeres:

$$ {\mathrm{F}}_{\mathrm{ROH}}={\displaystyle \sum {\mathrm{L}}_{\mathrm{ROH}}/\ {\mathrm{L}}_{\mathrm{AUTO}}} $$

The estimate of the total genome captured was 2 677 608 286 bp. F_ROH_ estimates inbreeding differently compared to the coefficients based on SNP-by-SNP indices F I, F II and F III as it considers only homozygous regions above a pre-defined length criterion (i.e. 1000 kb). Due to the F_ROH_ distribution in our sample we divided ROHs into two classes, below and above 1500 kb, and F_ROH_ was calculated overall, and for the two subclasses using the R statistics package [25]. The overall F_ROH_ was also tested for differences between cases and controls using a Student’s *t*-test.

## Results

After stringent quality control and exclusion of extreme population outliers the overall genetic matching was satisfying with a genomic control inflation factor at λ_gc_ = 1.00 within the prior GWAS, indicating that no population stratification was present [11].

### Proportion of total phenotypic variance explained by SNPs

The proportion of the total phenotypic variance explained by SNPs from the joint analysis transformed to the liability scale after Dempster and Lerner showed a value of 0.51 (SE 0.16 at *P* ≤ 1.97 × 10^−7^) [21]. After the exclusion of the regions covered by the previously identified TC risk SNPs the proportion of the total phenotypic variance explained by the so far unidentified SNPs was 0.33 (SE 0.15 at *P* ≤ 0.003). While most of variance explained by common SNPs for individual autosomes stayed constant, a major drop was detected for chromosome 2 encompassing *DIRC3* (from 0.11 to 0.03) and for chromosome 9 encompassing *FOXE1* (from 0.17 to 0.08).

### Genome-wide assessment of associations between homozygosity at individual SNPs and susceptibility to TC

Results of the association between homozygosity and the susceptibility to TC on a SNP-by-SNP basis are shown in Table 1. The FDR was calculated and controlled at an arbitrary level q* < 0.05, for which 34 SNP were significant [23]. Corresponding odds ratios (ORs) of the one-sided Fisher’s exact test to prove the hypothesis that increased homozygosity is associated with higher risk of TC showed a minimum of OR = 1.85 with a 95 % confidence interval of 1.23–3.41 for all SNPs in Table 1.

**Table 1:** Association between homozygosity and susceptibility to TC for individual SNPs

SNP	CHR	BP^a^	Cases AA/BB	Cases AB	Controls AA/BB	Controls AB	chi^2^	*P* ^b^	*q* ^*c^	Genes
rs4698482	4	16020011	519	116	274	157	44.43	2.62 × 10^−11^	1.40 × 10^−5^	LDB2
rs11688848	2	204624451	512	119	275	156	40.10	2.40 × 10^−10^	5.38 × 10^−5^	ICOS
rs9578483	13	22068754	543	97	296	135	39.66	3.01 × 10^−10^	5.38 × 10^−5^	FGF9, FTHL7
rs839509	2	212530542	497	126	270	160	37.09	1.12 × 10^−9^	0.0001	ERBB4, CPS1, hCG_1645016
rs2414003	15	48105489	514	122	280	151	33.90	5.77 × 10^−9^	0.0006	ATP8B4, SLC27A2
rs3096381	16	69875502	525	116	289	141	30.46	3.39 × 10^−8^	0.0028	FLJ11171, HYDIN, CALB2
rs630695	6	117359452	526	103	299	132	30.10	4.09 × 10^−8^	0.0028	RFXDC1, GPRC6A, VGLL2
rs938845	18	63860975	512	122	284	147	30.02	4.26 × 10^−8^	0.0028	NA
rs17797954	5	174303096	516	122	287	144	28.09	1.15 × 10^−7^	0.0068	DRD1
rs10961997	9	15361675	509	134	279	152	27.48	1.58 × 10^−7^	0.0083	SNAPC3
rs12126497	1	166939482	586	56	346	85	27.33	1.71 × 10^−7^	0.0083	DPT, XCL1
rs509716	6	131475408	532	113	297	134	26.90	2.13 × 10^−7^	0.0095	EPB41L2, AKAP7
rs6715968	2	229884476	484	141	272	159	25.75	3.86 × 10^−7^	0.0159	PID1, DNER
rs712082	1	222792683	545	98	311	120	25.32	4.83 × 10^−7^	0.0173	WDR26, AKR1B1P1
rs6440553	3	149713261	545	84	321	110	25.32	4.84 × 10^−7^	0.0173	RPL38P1
rs8043171	15	90065471	529	105	304	127	25.07	5.50 × 10^−7^	0.0184	SLCO3A1
rs12902263	15	69429108	556	87	321	110	24.77	6.44 × 10^−7^	0.0197	THSD4, hCG_2004593, NR2E3
rs10254361	7	119351441	522	116	296	135	24.72	6.62 × 10^−7^	0.0197	KCND2
rs11563992	7	27347461	507	115	294	136	24.16	8.86 × 10^−7^	0.0242	NA
rs7018634	9	20249528	538	100	310	121	24.11	9.05 × 10^−7^	0.0242	SLC24A2, SMNP
rs11169076	12	48261675	571	72	335	96	23.99	9.68 × 10^−7^	0.0247	MCRS1, FAM186B
rs1943939	18	69856260	556	62	342	89	23.22	1.43 × 10^−6^	0.0332	FBXO15,
rs12660310	6	167051901	503	120	292	139	23.18	1.46 × 10^−6^	0.0332	RPS6KA2, RNASET2
rs11204947	1	150484881	489	135	280	151	23.16	1.48 × 10^−6^	0.0332	HRNR, FLG,
rs3821310	2	74923771	581	61	345	85	23.06	1.56 × 10^−6^	0.0335	HK2, SEMA4F, POLE4
rs9407406	9	8229748	532	95	314	117	22.93	1.67 × 10^−6^	0.0345	C9orf123, PTPRD
rs2830028	21	26349119	493	133	282	148	22.64	1.94 × 10^−6^	0.0386	APP, GABPA, CYYR1
rs11151652	18	67133203	554	91	319	110	22.52	2.07 × 10^−6^	0.0397	CBLN2
rs10779770	1	12529312	537	97	314	117	22.42	2.18 × 10^−6^	0.0403	VPS13D, DHRS3
rs1508833	5	38050010	519	108	303	127	22.35	2.26 × 10^−6^	0.0404	GDNF, EGFLAM
rs554232	8	102533760	540	98	314	117	22.23	2.40 × 10^−6^	0.0408	NACAP1, GRHL2
rs2102727	8	53063166	502	133	285	146	22.21	2.43 × 10^−6^	0.0408	PCMTD1, ST18,
rs9379246	6	8777273	571	67	341	90	22.11	2.56 × 10^−6^	0.0416	HULC
rs7481683	11	8157762	454	174	252	179	21.98	2.75 × 10^−6^	0.0434	RIC3, LMO1

^a^Genome build hg18

^b^ *P* was calculated using a simple 2x2 chi^2^ test based on the number of homozygotes and heterozygotes at each SNP in cases and controls

^c^ *q* ^*^values representing the false discovery rate (FDR)

### Identification of ROHs and association between ROHs and TC susceptibility

We identified a total of 12 306 individual ROHs greater than 1000 kb across all 1080 individuals with 7523 ROHs in cases and 4783 ROHs in controls. On average 11.39 ROH segments with a total overall length of 22 980 kb per individual were detected. The average number of ROH segments per person in cases was 11.59 and in controls 11.09 (*P*_*diff*_ = 4.00 × 10^−2^), the total length of ROHs per person was 4761 kb higher in cases than in controls (*P*_*diff*_ = 1.95 × 10^−5^), and the average ROH length per person in kb was significantly higher in cases (1988 kb) than in controls (1788 kb) (*P*_*diff*_ = 3.29 × 10^−8^).

We extended the tests for association between ROHs and susceptibility to TC by categorizing the number of ROHs and the total length of ROHs in Mb by forming control groups of similar size. They were compared with the numbers of cases within the corresponding classes (Table 2). Cases had more ROHs and the total length of ROHs was also longer than in controls. (e.g. for entire data set >15 ROHs, OR = 1.55, *P* = 0.02; for >25.4 Mb, OR = 1.45, *P* = 0.03).

**Table 2:** Association between overall ROH and TC (min. 75 SNPs per ROH)

	Entire data set				
Number of ROH^a^	Cases	Controls	OR	95 % CI	*P*
< 10	204	152	1.00	Ref.	
10–12	145	88	1.22	0.87–1.72	0.23
13–15	170	127	0.99	0.73–1.36	0.98
> 15	130	64	1.55	1.05–2.18	0.02
Total length (Mb)					
< 14.1	153	117	1.00	Ref.	
14.1–19.4	156	114	1.04	0.74–1.47	0.79
19.4–25.4	163	107	1.16	0.82–1.64	0.38
> 25.4	177	93	1.45	1.02–2.06	0.03

^a^Cutoffs were chosen to produce approximately equal group sizes for cases and controls

For further association analysis 2262 consensus groups were formed, of which a total of 225 ROHs were identified, that fulfilled the criteria of identical start and end location and at least 75 consecutive homozygous SNPs [26]. An example for an overlapping region is given in the Additional file 1: Figure S1. None of the ROHs were associated with susceptibility to TC after correction for multiple testing. However, 16 ROHs were associated at a suggestive level (*P* < =0.05) (Table 3). None of them encompassed the centromeric regions.

**Table 3:** List of ROHs associated with TC

ROH	Chr.	Start – End (bp)^a^	Cases/controls	Chi^2^	*P* ^b^	*P* ^c^	iHS max^d^	F_st max_^e^	Fay and Wu’s H^f^	Genes^g^
ROH1	2	167204846–167895993	6 / 15	8,87	0.002	1.44 × 10^−4^	3.50	0.50	−74.64	XIRP2
ROH2	3	121016843–121689105	10 / 0	6,70	0.009	9.43 × 10^−6^	2.76	0.50	−37.03	GSK3B, FSTL1, LRRC58, GPR156
ROH3	10	44969326–45928700	5 / 11	5,63	0.01	6.12 × 10^−5^	1.85	0.35	−57.08	ALOX5, OR13A1, ANUBL1, CTGLF1, MARCH8, OR6D1P, FAM21C, CTGLF10P
ROH4	6	69734043–70381283	2 / 7	5,42	0.01	0.007	2.58	0.27	−31.48	BAI3^g^
ROH5	9	73966521–74829925	2 / 7	5,42	0.01	1.60 × 10^−12^	2.05	0.44	−19.76	ALDH1A1, ZFAND5, TMC1
ROH6	1	217208583–218034929	7 / 0	4,67	0.03	0.08	2.17	0.41	−55.42	LYPLAL1, ZC3H11B
ROH7	2	26036646–26765583	7 / 0	4,67	0.03	0.18	2.42	0.61	−64.28	HADHA, HADHB, OTOF, RAB10, SELI, C2orf39, CIB4, FAM59B, PPIL1P1, GPR113, C2orf70
ROH8	2	75174688–76481471	7 / 0	4,67	0.03	0.03	2.78	0.57	−66.54	C2orf3, MRPL19, FAM176A,
ROH9	1	177243354–178385972	6 / 0	4,00	0.04	1.67 × 10^−4^	2.67	0.38	−56.96	ABL2, SOAT1, NPHS2, CEP350, FAM20B, TOR1AIP1, IFRG15, TOR3A, C1orf125, FAM163A, TDRD5, TOR1AIP2
ROH10	2	112182736–113192306	6 / 0	4,00	0.04	0.02	2.54	0.41	−24.47	SLC20A1, MERTK, ANAPC1, POLR1B, CHCHD5, ZC3H8, TMEM87B, FBLN7, TTL, ZC3H6, RGPD8,
ROH11	2	113858688–114678121	6 / 0	4,00	0.04	0.83	2.37	0.50	−48.23	ACTR3, RABL2A, SLC35F5, RPL23AP7, CBWD2, RP11-395 L14.12, FOXD4L1, WASH2P
ROH12	4	181001922–181547116	6/ 0	4,00	0.04	0.33	2.29	0.53	−36.74	NA
ROH13	4	182307562–182564832	6 / 0	4,00	0.04	0.35	2.09	0.30	−31.12	hCG_2025798
ROH14	4	183848547–184539543	6 / 0	4,00	0.04	1.00 × 10^−8^	2.09	0.65	−56.96	DCTD, CLDN22, WWC2, C4orf38, FAM92A3, CLDN24
ROH15	9	107008151–108187183	6 / 0	4,00	0.04	0.51	2.75	0.58	−46.35	FKTN, TAL2, SLC44A1, GARNL2P, TMEM38B, FSD1L, DEPDC1P2
ROH16	15	96502627–98965249	6 / 0	4,00	0.04	3.01 × 10^−12^	3.09	0.65	−110.70	IGF1R, MEF2A, HSP90B2P, SYNM, LINS1, TTC23, LRRC28, LYSMD4, ADAMTS17, C15orf51, LASS3, FAM169B, FLJ42289, PRKXP1

^a^Chromosomal positions derived from the National Center for Biotechnology Information (NCBI), build 36, hg18

^b^Suggestive significance

^c^Significances for testing differences in homozygosity with H_0_ : μ_Cases_ = μ_Controls_; H_1_ : μ_Cases_ > μ_Controls_;

^d^Represents maximal absolute values for iHS, derived for CEU population ancestry from Haplotter, Phase II (http://haplotter.uchicago.edu/)

^e^Represents maximal values for F_st_, derived for CEU population ancestry from Haplotter, Phase II

^f^Represents minimum values for Fay and Wu’s H, derived for CEU population ancestry from Haplotter, Phase II

^g^in flanking region

Intriguingly, several recurrent ROHs harbor genes that have been associated with risk or progression of TC (Table 3). The first consensus region, located on chromosome 2, shows the strongest association with TC susceptibility (uncorrected P value = 0.002, ROH1 in Table 3). Six cases and 15 controls carried a ROH spanning this region of 79 homozygous SNPs. Another consensus region on chromosome 3 (ROH2) spans 672 kb and contains 98 SNPs. Genes and predicted transcripts include *GSK3B, FSTL1, LRRC58, GPR156*. A consensus region on chromosome 10 spanning 81 SNPs on a length of 959 kb (ROH3, *P* = 0.01) also hosts a considerable number of genes.

To scrutinize the significant ROH consensus regions, the average homozygosity for all SNP loci within a corresponding ROH was computed for cases and controls separately and tested for a difference with a one-tailed Student’s *t*-test (Table 3, column 9). Ten ROHs showed significant differences at *P* < 0.05 level, of which 6 had more cases than controls.

### Natural selection as a cause of ROHs

To assess the influence of selection on the recurrent ROH regions, we used the measures iHS, F_st_ and Fay and Wu’s H [28, 29, 31, 32]. Every recurrent ROH showed significant values for the three estimates (iHS >2.0, Fst >0.2 and Fay and Wu’s H < <−10; Table 3), except for ROH3, for which the iHS value was 1.85. This indicates that each of the 16 ROH regions might be the result of a selective sweep.

### Inbreeding and association between homozygosity and TC

We formally calculated the inbreeding coefficients (so called F I, F II and F III) after Yang et al. for all samples [22]. The means (SDs) for F I in cases and controls were 0.003 (0.01) and -0.0005 (0.006), respectively, and significantly different from each other (*P* = 2.94 × 10^−13^, by Student’s *t*-test). Thus, there was significant evidence that cases were more inbred than controls. This was supported by the inbreeding coefficient F III, which also differed significantly between cases and controls at *P* = 3.77 × 10^−6^ with cases being more inbred. The inbreeding coefficient F II was in cases 0.002 (0.01) and in controls 0.001 (0.007), but differences were not significant. Table 4 lists the P values for the test of true differences of F I, F II and F III between cases and controls separately for each chromosome. Chromosomes 2, 4, 5 and 8 were significantly different. For all chromosomes cases showed higher values for F I, F II and F III than controls.

**Table 4:** *P*-values for differences of inbreeding coefficients F I, F II and F III between cases and controls

CHR.	Length in BP	F I*	F II*	F III*
1	239482994	0.16	0.39	0.22
2	237975642	**0.006**	**0.01**	**0.01**
3	195481660	0.19	0.28	0.11
4	187415093	**0.0002**	**0.01**	**0.008**
5	175834594	**4.52 × 10** ^**−5**^	**0.03**	**0.0009**
6	165666786	0.09	0.50	0.64
7	154972229	**0.01**	0.72	0.36
8	141209234	**0.0004**	0.69	**0.04**
9	126549725	0.12	0.22	0.17
10	131868223	**0.02**	0.54	0.11
11	129246417	0.27	0.60	0.71
12	128925838	**0.01**	0.30	0.07
13	91046560	**0.01**	0.15	0.15
14	82460885	0.44	0.80	0.82
15	77621221	0.07	0.74	0.12
16	82341226	0.26	0.66	0.68
17	77544622	**0.02**	0.55	0.41
18	74113617	0.23	0.57	0.78
19	60064206	**0.02**	0.13	**0.04**
20	59665714	**0.002**	0.47	0.09
21	30132781	**0.001**	0.89	0.15
22	27989019	0.63	0.63	0.67

*bold values show significant differences between cases and controls at *P* < 0.05

When using a GLM with several covariates and regressing the explanatory variables F I, F II or F III on the disease status of the TC patient as the binary response (0/1), F I and F III remained significant at *P* = 0.003 with a positive effect estimate of 32.19 and 64.38, respectively. This results in an increasing slope of the regression line towards the diseased individuals. F II was also significant at *P* = 0.01.

**Fig. 1:**
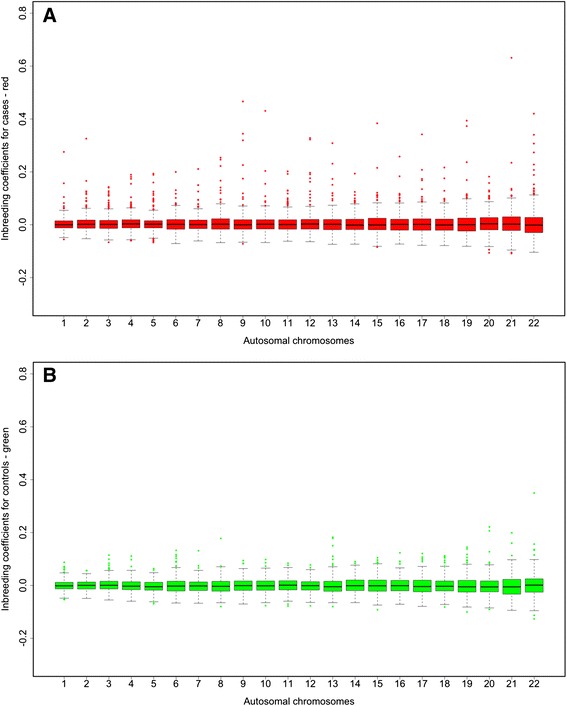
Variation of inbreeding coefficients among chromosomes for cases (**a**) and controls (**b**). The boxplot figure shows the means and variation of the inbreeding coefficient F I for autosomal chromosomes 1 to 22 for cases (*red*) and controls (*green*)

A more detailed overview on the characteristics of the inbreeding coefficient for cases and controls is demonstrated in Fig. 1, which shows the variation of the inbreeding coefficient between chromosomes. The mean is rather constant across the chromosomes but the variation is increasing from chromosome 1 to 22 while the length of the chromosomes in base pairs is decreasing (*r* = −0.80, *P* = 6.51 × 10^−6^).

**Fig. 2:**
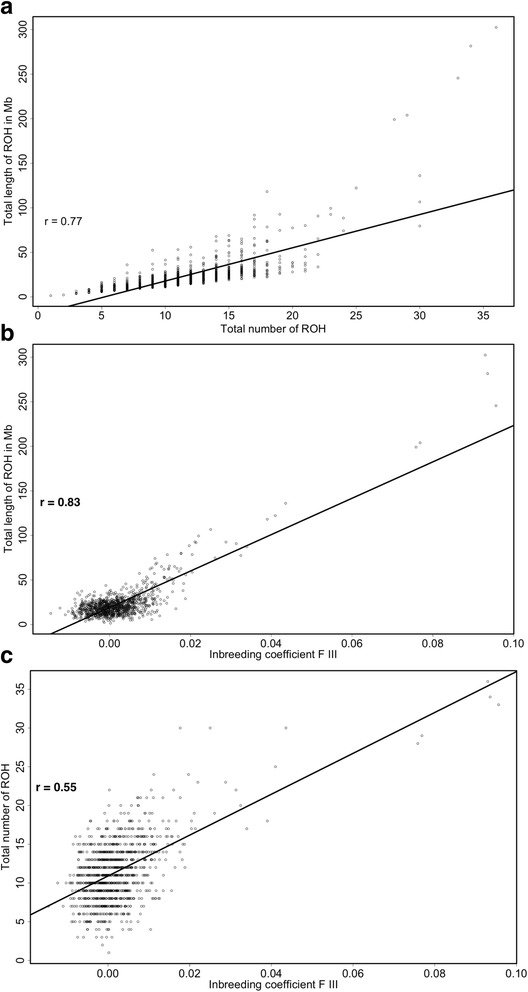
Pearson's correlation coefficients for different consanguinity measures. The total length of individual ROHs is highly correlated with the total number of ROHs per individual (*r* = 0.77, *P* < 2.20*10^-16^) (**a**). A high association is determined for the total length of ROHs in Mb and the individual inbreeding coefficient F III (*r* = 0.83, *P* < 2.20*10^-16^) (**b**), while the lowest association was determined for the total number of ROHs per individual and the individual inbreeding coefficient F III (*r* = 0.55, *P* < 2.20*10^-16^) (**c**)

Three additional associations for different consanguinity measures were tested (Fig. 2). The total length of individual ROHs was highly correlated with the total number of ROHs per individual (*r* = 0.77, *P* < 2.20 × 10^−16^). A significant association was also determined for the total length of ROHs per individual and the individual inbreeding coefficient F III (*r* = 0.83, *P* < 2.20 × 10^−16^) and for the total number of ROHs per individual and the individual inbreeding coefficient F III (*r* = 0.55, *P* < 2.20 × 10^−16^).

Finally, F_ROH_ was also 0.22 units of standard deviation SD (*P* = 1.95 × 10^−5^) higher in cases than controls. The correlation between the inbreeding coefficients and F_ROH_ were also highly significant (F I: *r* = 0.71, *P* = 2.20 × 10^−16^; F II: *r* = 0.72, *P* = 2.20 × 10^−16^; F III: *r* = 0.83, *P* = 2.20 × 10^−16^).

## Discussion

Based on our previous GWAS we showed here that the proportion of the total phenotypic variance in TC risk explained by all common SNPs is about 0.51. After correcting for identified TC risk loci about two-thirds of the genetic variance remain to be identified [11, 17]. This fact clearly shows the high influence of both the genetic factors and the environment on the susceptibility of TC. In the present study, we sought to find other genetic explanations than genes identified through previous GWASs that function in a co-dominant manner. The focus was shifted towards recessive inheritance. The current work is to our knowledge the first analysis of the influence of genomic homozygosity and genomic inbreeding on the susceptibility to TC.

Already the genome-wide SNP-by-SNP analysis showed significantly higher proportion of homozygous genotypes among the cases than controls. Further downstream analyses revealed significant differences between cases and controls in terms of the number and length of ROHs per person.

It is known that homozygosity can be caused by demographic events, consanguinity/inbreeding or selective pressure [33, 34]. Most of the ROHs in our study were rather short though. This excludes recent consanguinity as the cause of inbreeding. However, the significant genomic inbreeding coefficients point to a certain level of relatedness that might remain from distant consanguinity. All the ROHs of interest showed significant evidence for natural selection (iHS, F_st_, Fay and Wu’s H) [28]. The influence of selective pressure on the ROH length can therefore not be excluded.

The analysis of specific overlapping ROHs did not result in a genome-wide significance, however, several ROHs were matching with regions that contain genes related to TC susceptibility. The majority of overlapping ROHs was absent in controls. Homozygosity in these ROHs might have been disappeared over time due to recombination. Only for ROH1, ROH3, ROH4 and ROH5 we detected more controls than cases to be homozygous for an overlapping ROH region. One of these, ROH5 overlaps with long contiguous stretches of homozygosity from other studies [35, 36]. However, in 10 out of 16 consensus regions significantly higher amount of homozygous SNPs were observed among cases than among controls. Thus, the inheritance of recessive genes harbored in these regions might be possible.

Our study shows some evidence of an association between extended stretches of homozygosity and an increased TC risk. This result is not unexpected as several studies before have detected association between ROHs and cancer susceptibility [16].

The novel result of our study is the significant effect of genomic inbreeding among cases and its relevant effect on the development of the disease. The inbreeding coefficients F I, F II, and F III were significantly higher in cases than in controls, even after correcting for numerous covariates using GLM. Inbreeding is supposed to reduce fitness by causing an overabundance of homozygous loci and increasing the probability of deleterious rare alleles that lead to inbreeding depression [37]. As inbreeding is related to homozygosity, the chances of offspring being affected by recessive or deleterious traits are therefore increased [38]. In fact, the assumption that a higher level of inbreeding or increased homozygosity correlates with cancer incidence has been proven already before on the genomic level [16].

Even the results of the F_ROH_ support the higher inbreeding among cases compared with controls, although F_ROH_ is discarding SNPs in regions outside of ROHs that are below our stringent length criterion. The fact, that we found no significant differences among cases and controls in the mean sum of shorter ROHs but highly significant differences for the longer ROHs supports the view that the differences in ROH length longer than 20 Mb reflect effects of more recent consanguinity rather than LD pattern of ancient origin. It has been shown that consanguinity increased in Italy early in the 20th century and subsequently decreased. This has been explained by population growth in the early 20th century and changing demographics since then [39]. Another reason is the very large number of distantly related spouses in determining the population level of inbreeding [39]. With this source of a consanguineous population we had the unique opportunity to detect recessively inherited genomic regions for TC.

## Conclusion

We showed evidence for long ROHs to increase the risk of TC. Higher inbreeding among cases supports the existence of recessive alleles affecting TC risk. The genetic architecture of TC is highly supported by a genetic model, in which the variants of a complex disease are more likely to be rare than common. They are also likely to be numerous with highly polygenic architecture and of a small individual effect at the population level. If this view of the genetic architecture of common complex diseases is correct, then it would be important to consider inbreeding as a factor having an influence on the disease.

Supplementary information is available at the journals website.

## Additional file

Additional file 1: Figure S1. Example for recurrent ROHs in the telomeric region of chromosome 15 for 6 cases. (PDF 335 kb)

## Abbreviations

BP: base pair
CEU: Utah residents with ancestry from northern and western Europe
CHR: chromosome
CI: confidence interval
F: inbreeding coefficient
FDR: false discovery rate
F_st_: Fixation index
GCTA: genome-wide complex trait analysis
GLM: generalized linear regression model
GRM: genetic relationship matrix
GWAS: Genome-wide association study
iHS: integrated haplotype score
kb: Kilo-base pair
LD: linkage disequilibrium
Mb: Mega base pair
MLM: mixed linear model
OR: odds ratio
PTC: papillary thyroid cancer
REML: restricted maximum likelihood
ROH: runs of homozygosity
SD: standard deviation
SE: standard error
SNP: single-nucleotide polymorphism
TC: thyroid cancer
