# Supplementary material for: Runs of homozygosity and inbreeding in thyroid cancer
Source: BMC Cancer. 2016 Mar 16;16:227. doi: 10.1186/s12885-016-2264-7 (PMC4794977; doi:10.1186/s12885-016-2264-7)

Supplementary Figure 1: Example for recurrent ROHs in the telomeric region of chromosome 15 for 6 cases

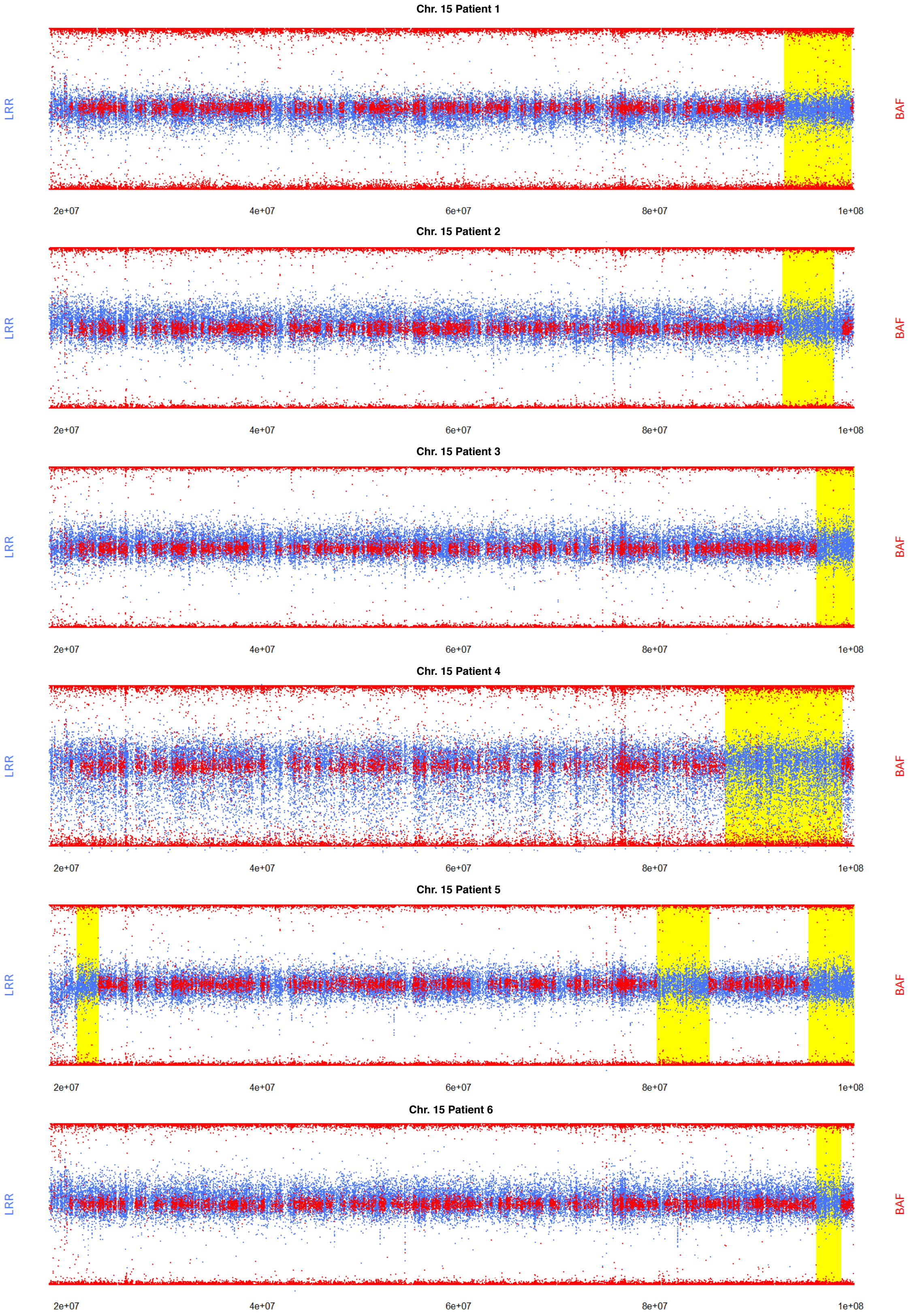

Supplement: Additional file 1: Figure S1. — Example for recurrent ROHs in the telomeric region of chromosome 15 for 6 cases. (PDF 335 kb) [file 12885_2016_2264_MOESM1_ESM.pdf]
